# Loss of *Arabidopsis β-COP* Function Affects Golgi Structure, Plant Growth and Tolerance to Salt Stress

**DOI:** 10.3389/fpls.2020.00430

**Published:** 2020-04-15

**Authors:** Judit Sánchez-Simarro, César Bernat-Silvestre, Fátima Gimeno-Ferrer, Pilar Selvi-Martínez, Javier Montero-Pau, Fernando Aniento, María Jesús Marcote

**Affiliations:** Departamento de Bioquímica y Biología Molecular, Estructura de Recerca Interdisciplinar en Biotecnología i Biomedicina (ERI BIOTECMED), Facultat de Farmàcia, Universitat de València, Valencia, Spain

**Keywords:** β-COP, coat protein I (COPI), coat protein II (COPII), Golgi apparatus, plant growth, salt stress, *Arabidopsis*

## Abstract

The early secretory pathway involves bidirectional transport between the endoplasmic reticulum (ER) and the Golgi apparatus and is mediated by coat protein complex I (COPI)-coated and coat protein complex II (COPII)-coated vesicles. COPII vesicles are involved in ER to Golgi transport meanwhile COPI vesicles mediate intra-Golgi transport and retrograde transport from the Golgi apparatus to the ER. The key component of COPI vesicles is the coatomer complex, that is composed of seven subunits (α/β/β’/γ/δ/ε/ζ). In *Arabidopsis* two genes coding for the β-COP subunit have been identified, which are the result of recent tandem duplication. Here we have used a loss-of-function approach to study the function of β-COP. The results we have obtained suggest that β-COP is required for plant growth and salt tolerance. In addition, β-COP function seems to be required for maintaining the structure of the Golgi apparatus.

## Introduction

The early secretory pathway involves bidirectional transport between the endoplasmic reticulum (ER) and the Golgi apparatus and is mediated by coat protein complex I (COPI)-coated and coat protein complex II (COPII)-coated vesicles. COPII vesicles are involved in ER to Golgi transport and their formation requires the sequential recruitment of five cytosolic components, the small GTPase SAR1 and the heterodimers SEC23/24 and SEC13/31 (Chung et al., 2016). COPI vesicles are involved in transport between Golgi cisternae (although its directionality is still a matter of debate) as well as in retrograde transport from the *cis*-Golgi to the ER. The basic component of the COPI coat is a complex (coatomer) composed of seven subunits (α/β/β’/γ/δ/ε/ζ) which are recruited *en bloc* from the cytosol onto Golgi membranes. The coatomer complex can be conceptually grouped into two subcomplexes, the B- (α/β’/ε) and F-subcomplex (β/δ/γ/ζ). The B-subcomplex has been proposed to function as the outer layer and the F-subcomplex as the inner layer of the COPI vesicle coat (Jackson, 2014). However, recent structural studies suggest that the COPI structure does not fit with the previously proposed model where the inner F-subcomplex is responsible of cargo selection while the outer B-subcomplex is responsible of membrane deformation, by analogy to coats based on clathrin/adaptor complexes (Dodonova et al., 2015). Following recruitment by the small GTPase ARF1, in its GTP-bound conformation, and cargo, COPI polymerizes on the membrane surface in such a way that COPI coat assembly depends on both membrane and cargo binding. However, much has yet to be learned about the specific functions played by the different subunits of the coatomer complex.

Genes encoding the components of the COPI machinery have been identified in plants (Robinson et al., 2007; Gao et al., 2014; Ahn et al., 2015; Woo et al., 2015). In *Arabidopsis*, all coatomer subunits (except γ- and δ-COP) have more than one isoform, in contrast to yeast, that contains only one isoform for every subunit, and mammals, which contain 2 isoforms of the γ- and ζ-COP subunits but only one for the others. In mammals and *Arabidopsis*, it has been proposed that different isoforms may be part of alternative coatomer complexes with different localization and perhaps different functions (Wegmann et al., 2004; Donohoe et al., 2007; Moelleken et al., 2007; Popoff et al., 2011; Gao et al., 2014). However, it has been recently reported that all of the isoforms of the mammalian COPI coat produce COPI-coated vesicles with strikingly similar protein compositions (Adolf et al., 2019).

The function of different plant COPI subunits has been studied by loss of function approaches. In *Nicotiana benthamiana* and tobacco BY-2 cells, depletion of β’, γ-, and δ-COP subunits suggest that the COPI complex is involved in Golgi maintenance and cell-plate formation, and that its prolonged depletion induces programmed cell death (Ahn et al., 2015). In *Arabidopsis*, knockout (KO) T-DNA mutants of the two α-COP subunit isoforms have been characterized. While the α*1-cop* mutant resembled wild type plants under standard growth conditions, the α*2-cop* mutant had defects in growth and the morphology of the Golgi apparatus was altered. A transcriptomic analysis of the α*2-cop* mutant showed upregulation of plant cell wall and endomembrane system genes, such as the COPII component *SEC31A* (Gimeno-Ferrer et al., 2017). Finally, knockdown of *Arabidopsis* ε-COP subunit isoforms has been reported to cause severe morphological changes in the Golgi apparatus and mislocalization of endomembrane proteins (EMPs) containing the KXD/E COPI interaction motif (Woo et al., 2015). In this manuscript, the function of the β-COP subunit has been studied for the first time in plants. We have found that loss of function of *Arabidopsis* β-COP affects Golgi structure, plant growth and tolerance to salt stress.

## Materials and Methods

### Plant Material and Stress Treatments

*Arabidopsis thaliana* ecotype Col-0 was used as wild type. The loss-of-function mutants *β1-cop* (SALK_002734) and *β2-cop* (SALK_017975C) were from the Salk Institute Genomic Analysis Laboratory^1^ and were obtained from the Nottingham Arabidopsis Stock Centre. *A. thaliana* plants were grown in growth chambers as previously described (Ortiz-Masia et al., 2007). To study whether salt tolerance was affected in the β-COP mutants, seeds of wild type (Col-0) and mutants were sown on Murashige and Skoog (MS) plates containing 100–150 mM NaCl. Plates were transferred to a controlled growth chamber after cold treatment in the dark for 3 days at 4°C. After 12 days, the rates of cotyledon greening were scored. To study mannitol (250–300 mM) and ABA (0.3–0.6 μM) tolerance the same protocol was used. Seeds harvested from Col-0 and mutant plants grown under the same conditions and at the same time were used. In some experiments, seeds of wild type (Col-0) and β-COP mutants were sown on MS plates without salt and grown for 4 days before being transferred to MS plates containing 160 mM NaCl. Three days after transplantation, the rates of cotyledon greening were scored.

### Electrolyte Leakage (EL)

Electrolyte leakage assays were performed as described previously (Jiang et al., 2017). Seeds of wild type (Col-0) and β-COP mutants were sown on MS plates without salt and grown for 4 days before being transferred to MS plates containing 135 mM NaCl. One day after transplanting, the seedlings were washed 3 times with deionized water to remove surface-adherent electrolytes and transferred to 50 mL tubes containing 25 mL of deionized water. Electrical conductivity (EC) was then measured as S_o_. The seedlings were gently shaken for 90 min, and the resulting EC was measured as S_1_. Then, the samples were autoclaved to release all electrolytes, cooled down and the final EC was measured as S_2_. Electrolyte leakage was measured as follows: EL = (S_1_-S_o_)/ (S_2_-S_o_) × 100 (%).

### Mutant Characterization

Mutant lines in a Col-0 background containing a T-DNA insertion (T-DNA mutants) were characterized by PCR. To obtain the amiRNA mutant, the β1/β2-directed amiRNA construct CSHL_0125A8 (Open Biosystems) was purchased from ABRC. Transformation of *Arabidopsis* with this construct was conducted according to the floral dip method (Clough and Bent, 1998). 10 transgenic plants containing the amiRNA construct were selected on half-strength MS medium containing appropriate antibiotics. All lines except one (line 5) showed a dwarf phenotype and gave a reduced number of seeds and only seeds from 5 lines could be collected. Transgenic lines 3, 5, and 10 that segregate 3:1 for antibiotic resistance were selected in the T2 generation. In lines 5 and 10, the T3 homozygous generation was used to characterize silencing by RT–qPCR as below. No T3 homozygous line with seeds was obtained from line 3 and T2 seedlings were used to characterize silencing. T2 and T3 lines were also characterized by PCR. The primers used for genotyping all mutants are included in Supplementary Table S1.

### Reverse Transcription Quantitative PCR (RT-qPCR)

Total RNA was extracted from seedlings using NucleoSpin RNA plant kit (Macherey-Nagel) and 3 μg of the RNA solution were reverse transcribed using the maxima first strand cDNA synthesis kit for quantitative RT-PCR (Fermentas) according to the manufacturer’s instructions. Quantitative PCR (qPCR) was performed by using a StepOne Plus machine (Applied Biosystems) with SYBR Premix Ex Taq TM (Tli RNaseH Plus) (Takara) according to the manufacturer’s protocol. Each reaction was performed in triplicate with 100 ng of the first-strand cDNA and 0.3 μM of primers for all the genes and 0.9 μM for *SEC31A* in a total volume of 20 μL. The specificity of the PCR amplification was confirmed with a heat dissociation curve (from 60 to 95°C). Relative mRNA abundance was calculated using the comparative Ct method according to Pfaffl (2004). Primers used for qPCR are listed in Supplementary Table S2.

### Isolation and Transformation of *Arabidopsis* Protoplasts

To obtain mesophyll protoplasts from *Arabidopsis* plants, the Tape-Arabidopsis Sandwich method was used, as described previously (Wu et al., 2009). Protoplasts were isolated from 4-week-old *Arabidopsis* rosette leaves of wild type and mutant plants. For transient expression, we used the polyethylene glycol (PEG) transformation method, as described previously (Yoo et al., 2007). Plasmids encoding marker proteins used were: ManI–GFP (Nebenführ et al., 1999) and calnexin-RFP (Künzl et al., 2016).

### Transient Transformation of *A. thaliana* Seedlings by Vacuum Infiltration

This protocol was adapted from the protocol described by Marion et al. (2008). For preparation of the *Agrobacterium* cultures used for agroinfiltration, the desired *Agrobacterium* (GV3101:pMP90 strain) was inoculated into 2.5 mL of Luria-Bertani (LB) growth medium containing the appropriate antibiotics. This pre-culture was grown overnight at 28°C in a shaking incubator and next day, 30 mL of LB containing the appropriate antibiotics were inoculated with 0.3 mL of the pre-culture and this culture was grown overnight. Once the *Agrobacterium* culture reached an OD around 2.2, cells were pelleted and resuspended with 2 mL of liquid MS medium. The suspension OD was measured again and the *Agrobacterium* suspension was diluted with the infiltration buffer (MS with 0.005% Silwet L-77^®^ and 200 μM acetosyringone) to have an OD of 2. Infiltration was performed by covering the 4–5 days old seedlings grown on MS 35 × 10 mm Petri dishes (4–6 dishes) with the *Agrobacterium* solution and by applying vacuum (300 mbar) with the help of a manometer twice for 1 min. Excess infiltration medium was subsequently removed and the plates were transferred to a culture room for 3 days. Healthy seedlings were selected and the cotyledons were analyzed by the abaxial side on the confocal microscope. Markers expressed using this system were ManI-YFP (Madison and Nebenführ, 2011), TIP1.1-GFP (Gattolin et al., 2011), and GFP-AGP4 (Martinière et al., 2012).

### Confocal Microscopy

Imaging was performed using an Olympus FV1000 confocal microscope^2^ with a 60× water lens. Fluorescence signals for GFP (488 nm/496–518 nm), YFP (514 nm/529–550 nm) and RFP (543 nm/593–636 nm) were detected. Sequential scanning was used to avoid any interference between fluorescence channels. Post-acquisition image processing was performed using the FV10-ASW 4.2 Viewer^®^.

### Transmission Electron Microscopy (TEM)

For electron microscopy, seedlings were grown on MS medium containing 1% agar, and the seedlings were harvested after 4 days. Chemical fixation of cotyledons was performed according to Osterrieder et al. (2010). Ultrathin (70 nm) sections were cut on a Microtome Leica UC6, stained with uranyl acetate and lead citrate and observed with a JEM-1010 (JEOL) transmission electron microscope. Post-acquisition image processing and quantification of the Golgi apparatus size was performed using ImageJ (v1.45) (Abramoff et al., 2004).

### Statistical Analysis

Differences in stress responses among β*1-cop*, β*2-cop*, and *amiR-*β*1/*β*2-cop* mutants compared to Col-0 were tested using a two sample *t*-test with unequal variances using Microsoft Excel 2013. For the analysis of Golgi length, data were analyzed using the software R, version 3.4.2 (Vienna, Austria). The lengths were compared using the Wilcoxon test; prior to this, the data were assessed for normality using the Shapiro–Wilk test.

### Genetic Variability of β-COP Genes

Number of copies of β-COP genes in different embriophyta genomes was obtained from EnsemblPlants^3^ (Vilella et al., 2009). Additionally, protein and coding sequences and number of synonymous (Ks) and non-synonymous (Ka) values for all orthologs were obtained from Ensembl database using their REST API. Moreover, *A. thaliana* sequences for all genes classified as paralogs were also obtained. Paralogs located at a physical distance of less than 2.5 Kb were naively identified as tandem duplications. Evolutionary distances among pair of paralogs and orthologs were estimated as transversion rates on fourfold degenerate synonymous sites (4DTv). The 4DTv was calculated using an in house Python script after aligning each pair of coding sequences using MACSE v.2.03 (Ranwez et al., 2011). Correction for multiple substitutions was applied (Tang et al., 2008). The maximum likelihood phylogenetic tree of the gene was inferred using IQTREE (Nguyen et al., 2015) from protein sequences. Best amino acid substitution model was JTT + I model selected based on the Bayesian information criterion (BIC). Branch support was obtained by bootstrap using an ultrafast method (Minh et al., 2013).

Genetic diversity of *A. thaliana* β-COP genes (at4g31480, at4g31490) was explored using the collection of single-nucleotide polymorphisms (SNPs) from the 1001 genomes database (1001 Genomes Consortium, 2016) which includes information for 1135 accessions. VCF files annotated using SNPEff were filtered to keep only SNPs with a moderate (e.g., non-synonymous amino acid substitution) or high impact changes (e.g., frameshifts or premature stop codons). In the case of non-synonymous substitutions putative effect was predicted using SNAP2 (Hecht et al., 2015), which takes into account both evolutionary information and structural features to assign a potential effect of changes. Nucleotide diversity (π) for each β-COP gene was calculated using custom Python scripts following VariScan implementation (Hutter et al., 2006); evolutionary distance between both genes was assessed using 4DTv. Additionally, nucleotide diversity was also calculated for a random sample of 5000 genes and genes classified as tandem duplications and 4DTv among tandemly duplicated genes.

## Results

### *Arabidopsis β-COP* Genes

**FIGURE 1:**
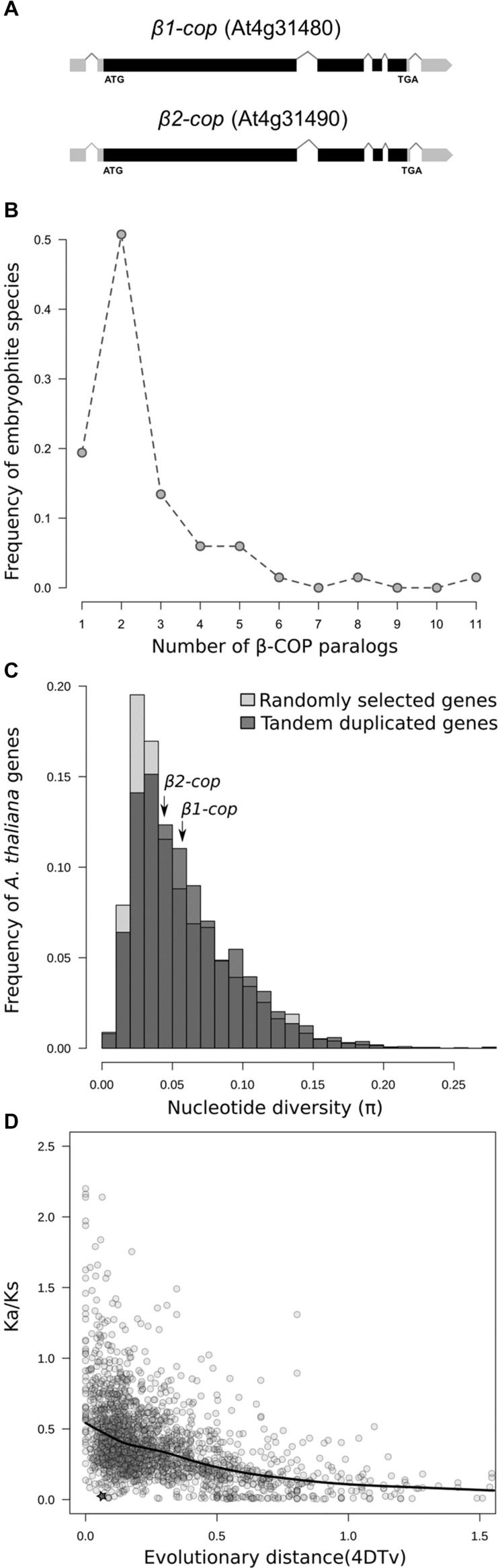
Genetic variability of β-COP genes. **(A)** Diagram of *β1-COP* and *β2-COP* genes. Black boxes represent coding regions and gray boxes represent 5′ UTR and 3′ UTR regions. **(B)** Number of β-COP paralogs among embryophyte species. **(C)** Histogram of the nucleotide diversity of 5000 randomly selected genes (light gray) and tandem duplicated genes (dark gray) in *A. thaliana.* Nucleotide diversity for β*1-COP* and β*2-COP* genes is marked with arrows. **(D)** Relationship between 4DTv distance and Ka/Ks ratio of pairs of tandemly duplicated genes in *A. thaliana*. Comparison between β*1-COP* and β*2-COP* is marked with a star. Line shows the local regression obtained by LOESS smoothing.

Two β-COP genes, *β1-COP* (At4g31480), and *β2-COP* (At4g31490) have been identified in *Arabidopsis*. They show high nucleotide and protein sequence identity (91 and 99%, respectively), identical number and position of introns (Figure 1A), and they exist in a tandem arrangement. Embryophytes tend to present at least two paralogs of β-COP genes, and in approximately 50% of species two copies are found (Figure 1B). Moreover, in 7 out of 72 plant species (*A. thaliana, Brassica napus, Leersia perrieri, Musa acuminata, Prunus persica, Tricicum aestivium*, and *Triticum dicoccoides*), at least two of the β-COP paralogs were found adjacent, likely being the result of tandem duplications, with an average distance among tandemly duplicated genes of 1.7 Kb (range from 71 to 4963 bp). The distribution of evolutionary distances measured as fourfold degenerate transversions (4DTv) among paralogs within species (Supplementary Figure S1A) suggests that evolutionary dynamics of paralogs is diverse, with some paralogs being the result of ancient duplications (4DTv > 0.5), while others seem to be the result of recent duplications (4DTv < 0.01). In the case of *A. thaliana*, both genes are the result of a recent tandem duplication (Supplementary Figure S1B) (Tiley et al., 2016). The genotyping of the 1135 *A. thaliana* accessions (1001 Genomes Consortium, 2016) resulted in 195 SNPs in β*1-COP* and 172 in *β2-COP.* Nucleotide diversity was 0.059 for *β1-COP* and 0.041 for *β2-COP*, which is in the average range of nucleotide diversity of either other *A. thaliana* genes or tandemly duplicated genes (Figure 1C). However, despite having a similar nucleotide diversity, the Ka/Ks ratio between both paralogs showed that both copies are under purifying selection (Ka/Ks = 0.06). Both copies tend to be more conserved than other *A. thaliana* tandemly duplicated paralogs (Figure 1D). These results could suggest the functional relevance of having two redundant gene copies. None of the SNPs resulted in a nonsense or frameshift mutation, and only 25 SNPs in *β1-COP* and 13 in *β2-COP* were non-synonymous changes. Of them, six and two, respectively, were classified as potentially having a deleterious effect. Interestingly, none of the accessions showed deleterious mutations in both genes simultaneously.

### Characterization of *β1-cop* and *β2-cop* Mutants

**FIGURE 2:**
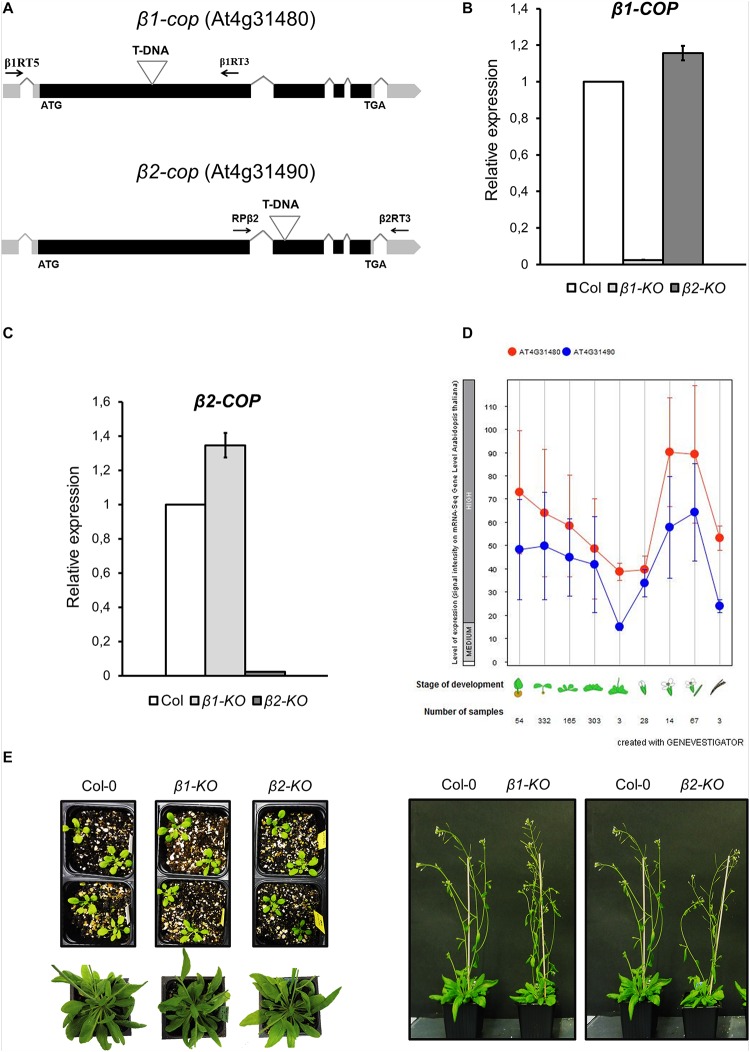
Characterization of *β1-cop* and *β2-cop* mutants. **(A)** Diagram of *β1-COP* and *β2-COP* genes and localization of the T-DNA insertion (triangle) in the mutants. Black boxes represent coding regions and gray boxes represent 5′ UTR and 3′ UTR regions. The positions of β1RT and β1RT3 primers and RPβ2 and β2RT3 primers used to genotype *β1-cop* and *β2-cop* mutants, respectively, are shown by arrows. **(B,C)** RT-qPCR analysis to show the absence of *β1-COP* **(B)** and *β2-COP* **(C)** mRNA in the *β1-cop* and *β2-cop* mutants, respectively. Total RNA was extracted from 4 days seedlings of the mutant and wild type (Col-0). The mRNA was analyzed by RT-qPCR with specific primers and normalized to UBQ10 expression (Supplementary Table S2). Results are from three biological samples and three technical replicates. mRNA levels are expressed as relative expression levels and represent fold changes of mutant/wild type. Values represent mean ± s.e.m. of the three biological samples. **(D)** Developmental stage-specific expression patterns of *β1-COP* and *β2-COP*. Seedlings, rosette leaves, floral organs and siliques are sequentially marked from left to right. “HIGH,” “MEDIUM,” and “LOW” expression were calculated by RNA-seq assay. The number of samples indicates RNA-seq gene expression data collected by GENEVESTIGATOR (www.genevestigator.com). **(E)** *β1-cop* and *β2-cop* mutants did not show any phenotype different from wild type.

To investigate the function of the two isoforms (β1-COP and β2-COP) of the β-COP subunit in *Arabidopsis*, T-DNA insertion mutants were identified and analyzed. These mutants were from the Salk collection and correspond to stock numbers SALK_002724 (*β1-cop* mutant) and SALK_017975C (*β2-cop* mutant) (Figure 2A). RT-PCR analysis confirmed that *β1-cop* and *β2-cop* mutants lacked the *β1-COP* and the *β2-COP* transcripts, respectively, and therefore, they can be considered loss-of-function mutants (Figures 2B,C). We next analyzed the expression levels of *β1-COP* and *β2-COP* in the *β1-cop* and *β2-cop* mutants. As shown in Figures 2B,C, *β1-cop* mutant showed around a 20% increase in the expression levels of *β2-COP* and *β2-cop* mutant showed around a 30% increase in the expression levels of *β1-COP*. Therefore, both genes seemed to be transcriptionally active as both *β1-COP* and *β2-COP* transcripts are detected and it is possible that loss of function of one isoform induces the expression of the other isoform. To investigate the relative expression of the two *β-COP* genes, we used the public available RNAseq expression database (Zimmermann et al., 2004) (GENEVESTIGATOR)^4^. As shown in Figure 2D, the expression pattern of *β1-COP* and *β2-COP* genes is similar, being higher the mRNA levels of *β1-COP*.

**FIGURE 3:**
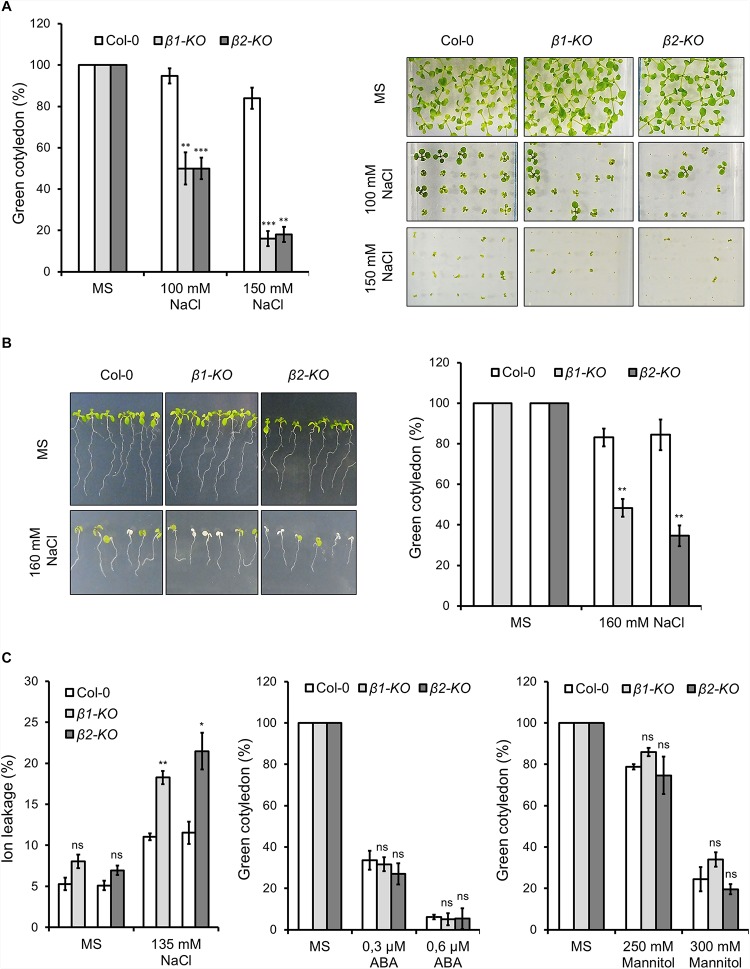
Phenotypic analysis of *β1-cop* and *β2-cop* mutants exposed to salt (NaCl), mannitol and ABA. **(A)** Wild type (Col-0) and *β1-cop* and *β2-cop* seeds were sown on 0.5 × MS as a control and 0.5 × MS supplemented with 100 mM or 150 mM NaCl. Left panel shows the percentage of seedlings with green cotyledons calculated after 12 days and are mean ± s.e.m. (*n* = 100) of four independent experiments. Right panel shows an image of a representative experiment. **(B)** Seeds of wild type (Col-0) and β-COP mutants were sown on MS plates without salt and grown for 4 days before being transferred to MS plates containing 160 mM NaCl. Three days after transplantation, the percentage of seedlings with green cotyledons was calculated and expressed as mean ± s.e.m. (*n* = 60) of three independent experiments (right panel). An image of a representative experiment is shown in the left panel. **(C)** Ion leakage of wild type and β-COP mutants one day after transplanting seedlings to MS plates containing 135 mM NaCl. Data show mean ± s.e.m of three independent experiments. **(D,E)** Wild type (Col-0) and *β1-cop* and *β2-cop* seeds were sown on 0.5 × MS or 0.5 × MS supplemented with ABA **(D)** or mannitol **(E)**. Data is the percentage of seedlings with green cotyledons calculated after 12 days and are mean ± s.e.m of three independent experiments (**D:** *n* = 60; **E:** *n* = 135). Statistical significance: ns, not significant; *p < 0.05; **p < 0.01; ***p < 0.001.

Neither *β1-cop* nor *β2-cop* mutants showed any phenotypic alteration under standard growth conditions (Figure 2E). We have previously shown that a mutant affecting 4 members of the p24 family, which are involved in COPI vesicle formation, showed enhanced sensitivity to salt stress (Pastor-Cantizano et al., 2018). Therefore, we tested whether *β1-cop* and *β2-cop* mutants were also sensitive to salt stress. To this end, seeds from wild type (Col-0), *β1-cop* and *β2-cop* mutants were sown in the presence of NaCl. As depicted in Figure 3A, both mutants were hypersensitive to salt stress, as shown by a drastic cotyledon greening reduction in the presence of 100-150 mM NaCl. To further investigate the salt sensitive phenotype, wild type, *β1-cop* and *β2-cop* mutants were first grown in normal growth medium and then transferred to plates containing 160 mM NaCl. As shown in Figure 3B, salt treatment caused a drastic reduction in cotyledon greening of *β1-cop* and *β2-cop* mutants, which confirms their sensitivity to salt stress. Finally, we also tested the effect of salt stress on ion leakage. As shown in Figure 3C, ion leakage was significantly increased in both *β1-cop* and *β2-cop* mutants in the presence of NaCl, which may reflect damage to cellular membranes in the mutants upon salt treatment. Since some salinity responses are regulated by abscisic acid (ABA) (Zhu, 2016), *β1-cop* and *β2-cop* mutants were also sown in the presence of ABA. However, similar sensitivity to ABA was detected in the mutants and wild type (Figure 3D). *β-cop* mutants were also treated with mannitol to test osmotic stress tolerance. Figure 3E shows that none of the mutants were hypersensitive to mannitol, suggesting that the NaCl sensitivity observed in the mutants is not due to osmotic stress.

### *AmiR-β1/β2-cop* Plants Display a Dwarf Phenotype and Enhanced Sensitivity to Salt Stress

**FIGURE 4:**
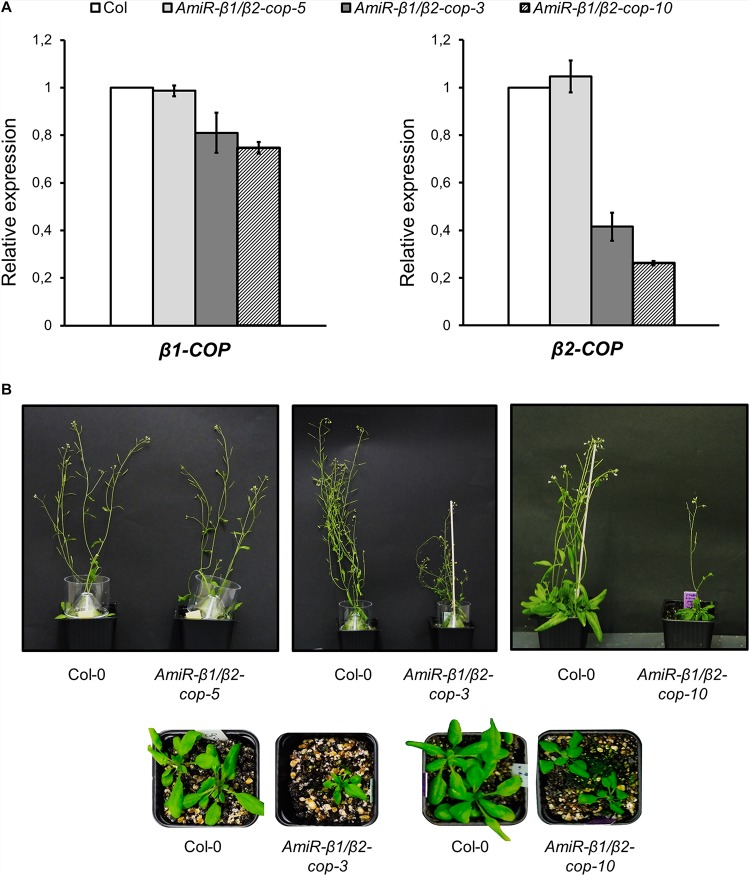
Characterization of *amiR-β1/β2-cop* mutants. **(A)** RT-qPCR analysis to show the silencing of *β1-COP* and *β2-COP* in the *amiR-β1/β2-cop* lines. Total RNA was extracted from 4 days seedlings of the amiRNA lines *amiR-β1/β2-cop-3*, *amiR-β1/β2-cop-5, amiR-β1/β2-cop10* and wild type (Col-0). The mRNA was analyzed by RT-qPCR with specific primers and normalized to UBQ10 expression (Supplementary Table S2). Results are from three biological samples and three technical replicates. mRNA levels are expressed as relative expression levels and represent fold changes of mutant/wild type. Values represent mean ± s.e.m of the three biological samples. **(B)** Phenotypes of amiRNA lines and wild type (Col-0) plants. Line 5: 36 day-old plants. Line 3: 26 (lower panel) and 50 day-old-plants (upper panel). Line 10: 26 (lower panel) and 36 day-old plants (upper panel).

To further investigate the function of the two isoforms of β-COP, we decided to knock down the expression of both genes. Since both genes are in tandem in chromosome 4, it was not possible to generate the double KO mutant. In order to simultaneously silence both *β-COP* genes, we used the artificial microRNA (amiRNA) CSHL_0125A8 (Open Biosystems). The amiRNA that we here called *amiR-β1/β2-COP* is targeted to a common region at the middle of the first exon of both *β-COP* genes. *A. thaliana* transgenic lines were generated by transformation with *amiR-β1/β2-COP*. A total of 10 independent lines (lines 1–10) were selected. All lines except one (line 5) showed a dwarf phenotype and gave a reduced number of seeds. The expression levels of both *β1-COP* and *β2-COP* genes were analyzed in three of these lines (lines 3, 5, and 10) by RT-qPCR. A reduction of both *β-COP* transcript levels was observed in the *amiRb1/β2-cop-3* and *amiR-β1/β2-cop-10* lines but not in *amiR-β1/β2-cop-5* (line 5) compared to the expression of wild type (Col-0) seedlings (Figure 4A). In contrast to the normal growth of *β1-cop* and *β2-cop* mutants, the *amiR-β1/β2-cop-3 and amiR-β1/β2-cop-10* plants exhibited a dwarf phenotype with reduced rosette leaf size and plant height (Figure 4B). Interestingly, the amiR-*β1/β2-cop-5*, which had no reduction in the expression levels of *β1-* and *β2-COP*, did not show any phenotypic alteration (Figure 4B), suggesting that the dwarf phenotype of the *amiR-β1/β2-cop-3 and amiR-β1/β2-cop-10* plants is a consequence of the reduced expression levels of β-COP. For the following experiments the *amiR-β1/β2-cop-10* line was used.

**FIGURE 5:**
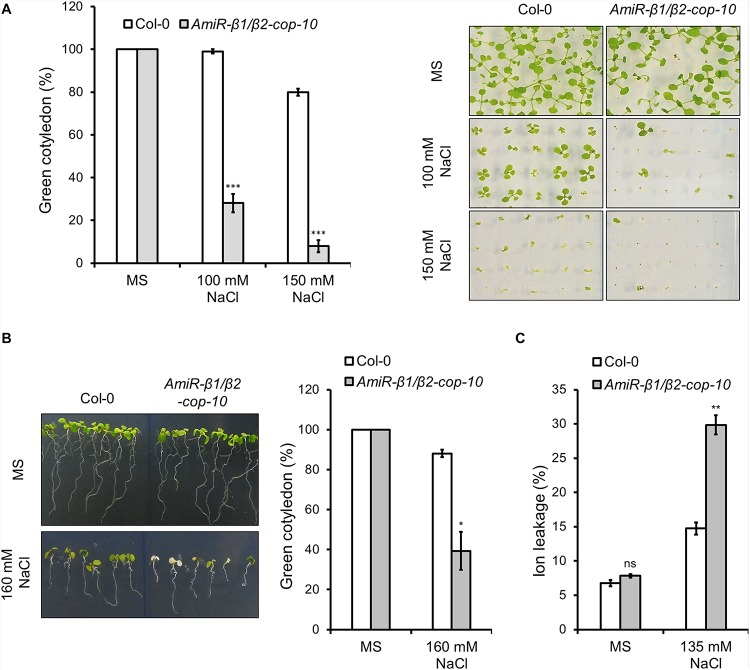
Phenotypic analysis of *amiR-β1/β2-cop* exposed to salt (NaCl). **(A)** Wild type (Col-0) and *amiR-β1/β2-cop* seeds were sown on 0.5 × MS for control conditions and 0.5 × MS supplemented with 100 mM NaCl and 150 mM NaCl in Petri plates. The percentage of seedlings with green cotyledons was calculated after 12 days. Data are mean ± s.e.m. (*n* = 100) of four independent experiments. **(B)** Seeds of wild type (Col-0) and the *amiR-β1/β2-cop* mutant were sown on MS plates without salt and grown for 4 days before being transferred to MS plates containing 160 mM NaCl. Three days after transplantation, the percentage of seedlings with green cotyledons was calculated and expressed as mean ± s.e.m. (*n* = 60) of three independent experiments (right panel). An image of a representative experiment is shown in the left panel. **(C)** Ion leakage of wild type and the *amiR-β1/β2-cop* mutant 1 day after transplanting seedlings to MS plates containing 135 mM NaCl. Data show mean ± s.e.m of three independent experiments. Statistical significance: ns, not significant; *p < 0.05; **p < 0.01; ***p < 0.001.

We next investigated whether the *amiR-β1/β2-cop-10* mutant had enhanced sensitivity to salt stress, as the single KO mutants. As shown in Figure 5, the *amiR-β1/β2-cop-10* mutant was also hypersensitive to salt stress, either by sowing the seeds directly in NaCl-containing medium (Figure 5A) or else by growing the seeds in normal growth medium before transfer to salt-containing plates (Figure 5B). We also found that the *amiR-β1/β2-cop-10* mutant had an increased ion leakage in the presence of salt, compared to wild-type plants (Figure 5C), as it was the case with the single *β1-cop* and *β2-cop* mutants.

### *AmiR-β1/β2-cop* Plants Show an Alteration in the Structure of the Golgi Apparatus

**FIGURE 6:**
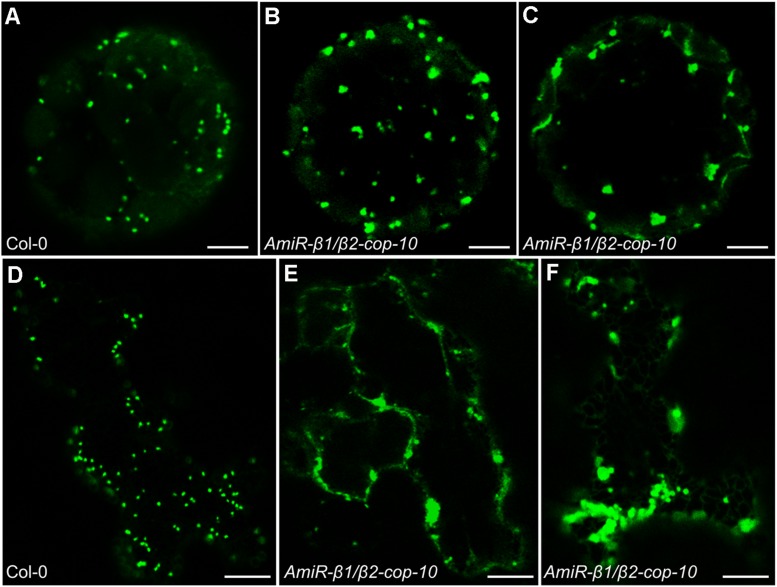
Localization of ManI-GFP in the *amiR-β1/β2-cop* mutant. **(A–C)** Transient gene expression of ManI-GFP in *Arabidopsis* protoplasts obtained from wild type (Col-0) or *amiR-β1/β2-cop* plants grown 4 weeks in soil. Scale bars: 5 μm. **(D–F)** Transient gene expression of ManI-YFP in *Arabidopsis* seedlings of wild type (Col-0) or *amiR-β1/β2-cop*. Scale bars: 10 μm.

As COPI vesicles have a main role in intra-Golgi transport and in retrograde transport from the *cis*-Golgi to the ER, we examined whether depletion of the β-COP subunit had an effect on the morphology of the Golgi apparatus. For this purpose, we first used transient expression experiments in *Arabidopsis* protoplasts, using a marker of the *cis* side of the Golgi apparatus, Mannosidase I–GFP (Nebenführ et al., 1999). ManI–GFP showed the typical punctate pattern characteristic of normal Golgi stacks in protoplasts obtained from wild type plants (Figure 6A). However, in one third of the protoplasts from the *amiR-β1/β2-cop* mutant ManI–GFP localized to clusters of punctate structures (Figures 6B,C), which suggests an alteration in the organization of the Golgi apparatus in this mutant. To confirm this Golgi phenotype, we also performed transient expression experiments in *Arabidopsis* seedlings, using the same Golgi marker. As shown in Figures 6E–F, ManI-YFP was also partially found in clusters of punctate structures in the *amiR-β1/β2-cop* mutant, in contrast to the normal punctate pattern observed in wild-type seedlings (Figure 6D). In contrast, no significant change was observed in the localization of other subcellular marker proteins in the *amiR-β1/β2-cop* mutant when compared with wild-type plants, including calnexin-RFP (endoplasmic reticulum) (Supplementary Figure S2), as well as two post-Golgi markers, TIP1.1-GFP (tonoplast) and the arabinogalactan protein GFP-AGP4 (plasma membrane) (Supplementary Figure S3).

**FIGURE 7:**
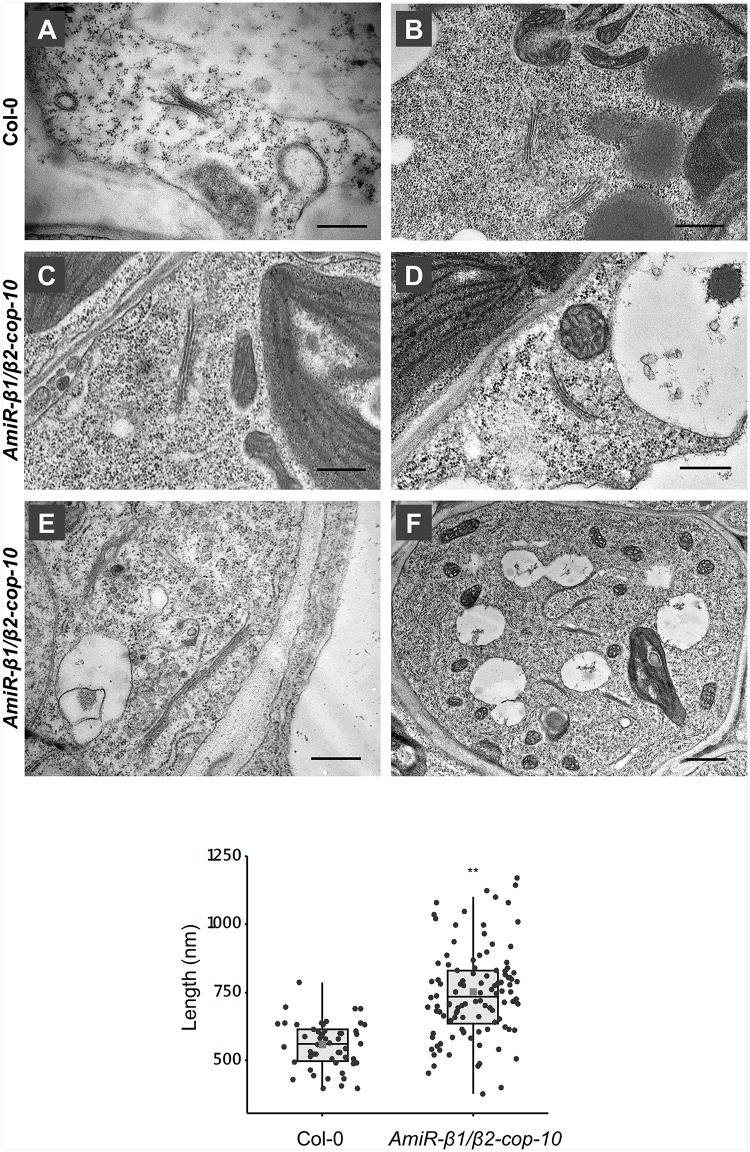
Ultrastructural analysis of the *amiR-β1/β2-cop* mutant. Chemically fixed cotyledon cells from 4-day-old seedlings of wild type (Col-0) **(A,B)** and the *amiR-β1/β2-cop* mutant **(C–F)**. Scale bars: **(A–E)** – 500 nm; **(F)** – 1 mm. **(G)** Quantification of the length of the Golgi apparatus in wild-type (Col-0) cells and in the *amiR-β1/β2-cop* mutant. *n* = 52 (Col-0) and 111 (*amiR-β1/β2-cop*). Golgi length was higher in the *amiR-β1/β2-cop* mutant compared with wild-type (Col-0). Statistical significance: **p < 0.01.

To gain insight into the defects observed in the *amiR-β1/β2-cop* mutant at the ultrastructural level, we performed transmission electron microscopy (TEM) analysis of ultrathin sections of seedlings processed by chemical fixation. As shown in Figure 7, the *amiR-β1/β2-cop* mutant showed enlarged Golgi stacks, with a length significantly higher than that observed in wild type cotyledons, although there was no obvious change in the number of cisterna. In some cases, these structures looked as if they were the result of the fusion between two adjacent stacks (Figure 7E). Therefore, the *amiR-β1/β2-cop* mutant seems to have an alteration in the structure of the Golgi apparatus.

### *β-cop* Mutants Show Increased Expression of *SEC31A*, That Encodes One Subunit of the COPII Coat

**FIGURE 8:**
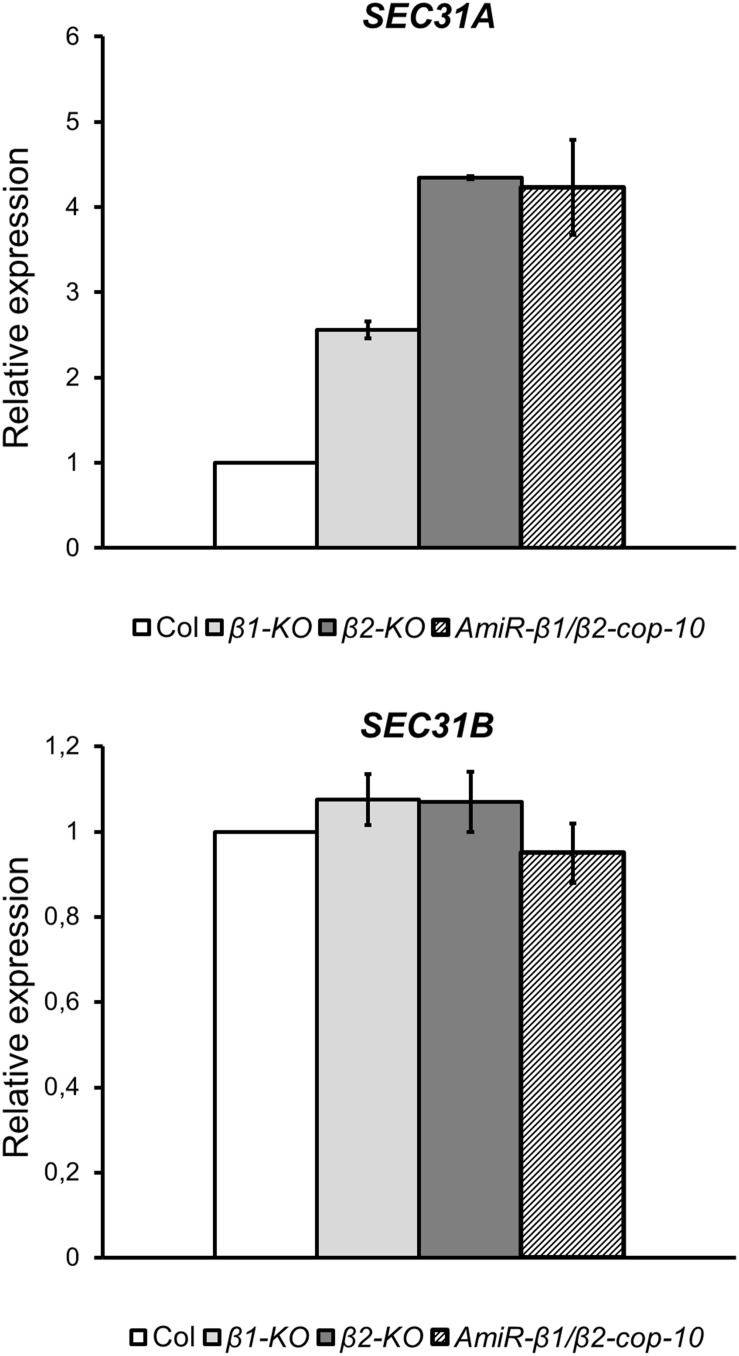
The *β1-cop*, *β2-cop* and *amiR-β1/β2-cop* mutants show upregulation of the COPII subunit SEC31A gene. Expression of *SEC31A* and *SEC31B* was analyzed by RT-qPCR. Total RNA was extracted from 4-day-old seedlings of wild type (Col-0) and the *β-cop* mutants. The mRNA was analyzed by RT-qPCR with specific primers (Gimeno-Ferrer et al., 2017) and normalized to UBIQ10 gene expression (Supplementary Table S2). mRNA levels are expressed as relative expression levels and represent fold changes of mutant over wild type. Values represent mean ± s.e.m. of three biological samples.

SEC31 is a component of the coat protein complex II (COPII) which promotes the formation of transport vesicles from the endoplasmic reticulum (ER). The *Arabidopsis* genome encodes two SEC31 isoforms, SEC31A (At1g18830) and SEC31B (At3g63460) (Chung et al., 2016). We have found previously that a mutant in one of the isoforms of the α-COP subunit of COPI, *α2-cop*, showed a strong up-regulation of *SEC31A*. This up-regulation of *SEC31A* seems to be specific for this particular COPII subunit as the expression other COPII subunit genes did not change. Therefore, we analyzed whether this was also the case for the *β-cop* mutants. As shown in Figure 8, both *β1-cop* and *β2-cop* mutants, as well as *amiR-β1/β2-cop-10* showed increased expression of *SEC31A*. In contrast, *SEC31B* was not up regulated in any of the mutants (Figure 8).

## Discussion

COPI-coated vesicles have been shown to be involved in trafficking within the Golgi and from the Golgi to the ER (Cosson and Letourneur, 1994; Letourneur et al., 1994). COPI is a heptameric protein complex composed of α, β, β’, δ, ε, γ, and ζ subunits (Waters et al., 1991) that is recruited onto Golgi membranes in the presence of the small GTP-binding protein ARF1. In this work, depletion of the β-COP subunit has been studied for the first time in plants. Embryophytes tend to present at least two paralogs of β-COP genes, and in *A. thaliana* the two genes coding for β-COP subunits (*β1-COP and β2-COP*) are the result of recent tandem duplication. These two genes seem to be evolutionarily conserved and none nonsense or frameshift naturally occurring mutants have been observed. Here we have shown that single null T-DNA insertion mutants of *β1-COP* and *β2-COP* show the same phenotype as wild type plants under standard growth conditions. One possible explanation is that loss of function of one isoform induces the expression of the other isoform, as shown in Figures 2B,C, which may be sufficient for normal growth under standard growth conditions, but not to deal with stress conditions. Indeed, both mutants showed enhanced sensitivity to salt stress. Interestingly, *amiR-β1/β2-cop* plants that have reduced levels of both *β1-COP* and *β2-COP* showed defects of growth in addition to enhanced sensitivity to salt stress. According to qPCR analysis, the mRNA levels of *β1-COP* and *β2-COP* were reduced by 20–25% and 60–75%, respectively. The different growth phenotype among the two single null mutants and the *amiR-β1/β2-cop* plants may be explained by the fact that plant amiRNAs have also been shown to have an effect at the translational level (Yu and Pilot, 2014). When grown in the presence of NaCl, the *amiR-β1/β2-cop* mutant was even more sensitive than the single KO mutants. This could be explained, as it has been suggested above, if this mutant has higher β-COP depletion. Unfortunately, the β-COP protein levels could not be tested as we have not found any β-COP antibodies that recognized *Arabidopsis* β-COP. Expression of both isoforms may be important to respond to salt stress, probably to support the trafficking of ion channels or transporters. Indeed, β-COP protein has been shown to regulate the surface expression of several ion channels in mammalian cells (Ryu et al., 2019). In fact, disorders in trafficking of plasma membrane and vacuole Na^+^/H^+^ antiporters in *Arabidopsis* may cause hypersensitivity to salt stress (Hamaji et al., 2009; Kim and Bassham, 2011; Kim et al., 2017). On the other hand, β-COP mutants also showed a partial mislocalization of mannosidase I, a specific membrane marker for plant *cis*-Golgi (Nebenführ et al., 1999), that is the Soybean ortholog of Arabidopsis α-1, 2-mannosidases MNS1 and MNS2 (Kajiura et al., 2010). These two redundant class I α-mannosidases cleave three α-1,2-mannosyl residues in ER-derived glycoproteins, to generate the substrate for the subsequent addition of GlcNAc. Interestingly, it has been described that MNS1/2-mediated mannose trimming of N-glycans is crucial in modulating glycoprotein abundance to withstand salt stress (Liu et al., 2018).

Transient expression experiments in seedlings and protoplasts (using a specific Golgi marker), together with electron microscopy analysis of the *amiRβ1/β2-cop* mutant showed morphological changes of the Golgi apparatus. This indicates that β-COP is important for the maintenance of Golgi structure, as it has been shown for α2-COP, β′-COP, and ε-COP subunits (Ahn et al., 2015; Woo et al., 2015; Gimeno-Ferrer et al., 2017). At the CLSM, Golgi punctae appeared frequently clustered, while at the electron microscope the Golgi apparatus appeared enlarged, with a length significantly higher than that in wild-type plants. It has been previously described that COPI-coated vesicles are essential for Golgi homeostasis. Concerning the β-subunit, knockdown of β-COP in HeLa cells was shown to produce an increase in Golgi volume and a fragmented Golgi (Guo et al., 2008). The apparent volume increase of Golgi markers was proposed to reflect defects in COPI-mediated membrane retrieval from the Golgi to the ER. Indeed, the surface area of Golgi cisternae depends on the ratio of membrane input and output to the Golgi apparatus along different trafficking routes (Sengupta and Linstedt, 2011). In this respect, inhibition of Golgi-to-ER transport by knockdown of Scyl1 (a high affinity-binding partner for COPI coats involved in the regulation of COPI-mediated retrograde trafficking) led to an expanded but intact Golgi in Hela cells (Burman et al., 2010). Therefore, the increased length of Golgi stacks observed in the *amiRb1/β2-cop* mutant may be the result of defective membrane recycling from the Golgi apparatus. Alternatively, the defect in β-COP might facilitate membrane fusion between Golgi stacks. The biogenesis of the Golgi ribbon in mammalian cells starts with clustering of Golgi stacks followed by tethering and homotypic fusion of Golgi stacks into a continuous ribbon (Nakamura et al., 2012). Although the plant Golgi apparatus does not form ribbon-like structures (Ito et al., 2014), it is still possible that Golgi stacks may eventually undergo fusion events under certain circumstances, such as COPI depletion. Indeed, Golgi cisternae have been shown to undergo homotypic fusion in *Saccharomyces cerevisiae* (Bhave et al., 2014), although Golgi apparatus in these cells consist in dispersed cisternae in the cytoplasm, without stacks and ribbon structures (Ito et al., 2014). In *Saccharomyces cerevisiae*, depletion of ARF1, which is involved in COPI vesicle formation, also led to Golgi enlargement, which was proposed to be the result of altered dynamics of cisternal maturation (Bhave et al., 2014).

Knockout of the α2-COP isoform of *Arabidopsis* α-COP subunit also caused an alteration in Golgi morphology. However, the Golgi phenotype observed upon α2-COP knockout consisted of a reduced number of cisternae per Golgi stack and many abnormal vesicle clusters around the Golgi remnants (Gimeno-Ferrer et al., 2017). This Golgi phenotype was similar to that obtained upon silencing of ε-COP in *Arabidopsis* and β’-COP in *N. benthamiana* (Ahn et al., 2015; Woo et al., 2015). This is different to the Golgi phenotype obtained upon silencing of β-COP isoforms, which rather produced longer Golgi stacks (this manuscript). This might be related with the fact that β-COP belongs to the F-subcomplex of coatomer, different from the B-subcomplex (including α, β′, and ε-COP subunits), although further work is required to dissect the function of specific COPI subunits in plants.

Finally, all three mutants of β-COP showed induction of the COPII subunit isoform *SEC31A*, but not that of *SEC31B*. This specific induction of *SEC31A* was also observed in the α2-COP mutant (Gimeno-Ferrer et al., 2017) as well as in a quadruple mutant affecting p24 family proteins, which are essential in COPI vesicle formation (Pastor-Cantizano et al., 2018). Altogether, there seems to be a direct correlation between alterations in COPI function and changes in the expression of the SEC31A subunit of the COPII complex, which is involved in the anterograde ER to Golgi transport.

## Data Availability Statement

All datasets generated for this study are included in the article/Supplementary Material.

## Author Contributions

MM and FA: conceptualization, writing – original draft, supervision, project administration, and funding acquisition. JS-S, FG-F, PS-M, JM-P, CB-S, MM, and FA: investigation. MM, JM-P, JS-S, and FA: writing – review and editing.

## Conflict of Interest

The authors declare that the research was conducted in the absence of any commercial or financial relationships that could be construed as a potential conflict of interest.

## Footnotes

1 http://signal.salk.edu/cgi-bin/tdnaexpress

2 http://www.olympus.com/

3 http://plants.ensembl.org/

4 www.genevestigator.com
