# Supplementary material for: Loss of Arabidopsis β-COP Function Affects Golgi Structure, Plant Growth and Tolerance to Salt Stress
Source: Front Plant Sci. 2020 Apr 15;11:430. doi: 10.3389/fpls.2020.00430 (PMC7175232; doi:10.3389/fpls.2020.00430)
Supplement: Supplementary file 1 [file Data_Sheet_1.pdf]

Loss of *Arabidopsis β-COP* function affects Golgi structure, plant growth and tolerance to salt stress

Judit Sánchez-Simarro, César Bernat-Silvestre, Fátima Gimeno-Ferrer, Pilar Selvi-Martínez, Javier Montero-Pau, Fernando Aniento and María Jesús Marcote

SUPPLEMENTAL MATERIAL

Table S1. Genotyping primers used for identification of *β–cop* mutants by conventional PCR.

Primer	Target	Sequence (5'→3')	Tm (ºC)
ami5	*amiRNA*	ATATAAGGAAGTTCATTTCATTTGGAG	55'1
ami3	*amiRNA*	GGATCCGCAATTAACCCTCACTA	59'1
β1RT5	*β1-COP*	GGATCCTCACCTGACGAATCCACCACC	63'9
β1RT3	*β1-COP*	GGATCCAATCCTCTCATACGAATCAT	56'2
β2RT3	*β2-COP*	ACGACTCTTCTCTCCATGTAGT	54'7
RPβ2	*β2-COP*	AGATATGGTTGGAATCCTGCC	54'8

Table S2. qPCR primers used for characterization of *β–cop* mutants.

Primer	Target	Sequence (5'→3')	Tm (ºC)
qβ1IF	*β1-COP* At4g31480	CATAAGGATAAGGAG CAAGACGCAA	64
qβ1IR	*β1-COP* At4g31480	CACTGTGAGAAATCA TCGGTATGGTA	65
qβ2F1	*β2-COP* At4g31490	TCTTCCTCAACCGGCT GTCTT	61
qβ2R1	*β2-COP* At4g31490	CGCGTTCAACAGTTCA GTCTCCT	65
Sec31AF	*SEC31A* At1g18830	AACGTGATTTTGGTGC AGCGTTA	57.9
Sec31AR	*SEC31A* At1g18830	TGGAAGCCAAGAACT GCACTCATC	59.5
Sec31BF	SEC31B At3g63460	CAGCAGCTGGACCCA TAGGATTTAC	53.9
Sec31BR	SEC31B At3g63460	GCTGTGTTGGAGGACT TGCTGGTTG	62.2
UBQ10F	*UBQ10* At4g05320	GGCCTTGTATAATCCC TGATGAATAAG	55.7
UBQ10R	*UBQ10* At4g05320	AAAGAGATAACAGGA ACGGAAACATAGT	56.1

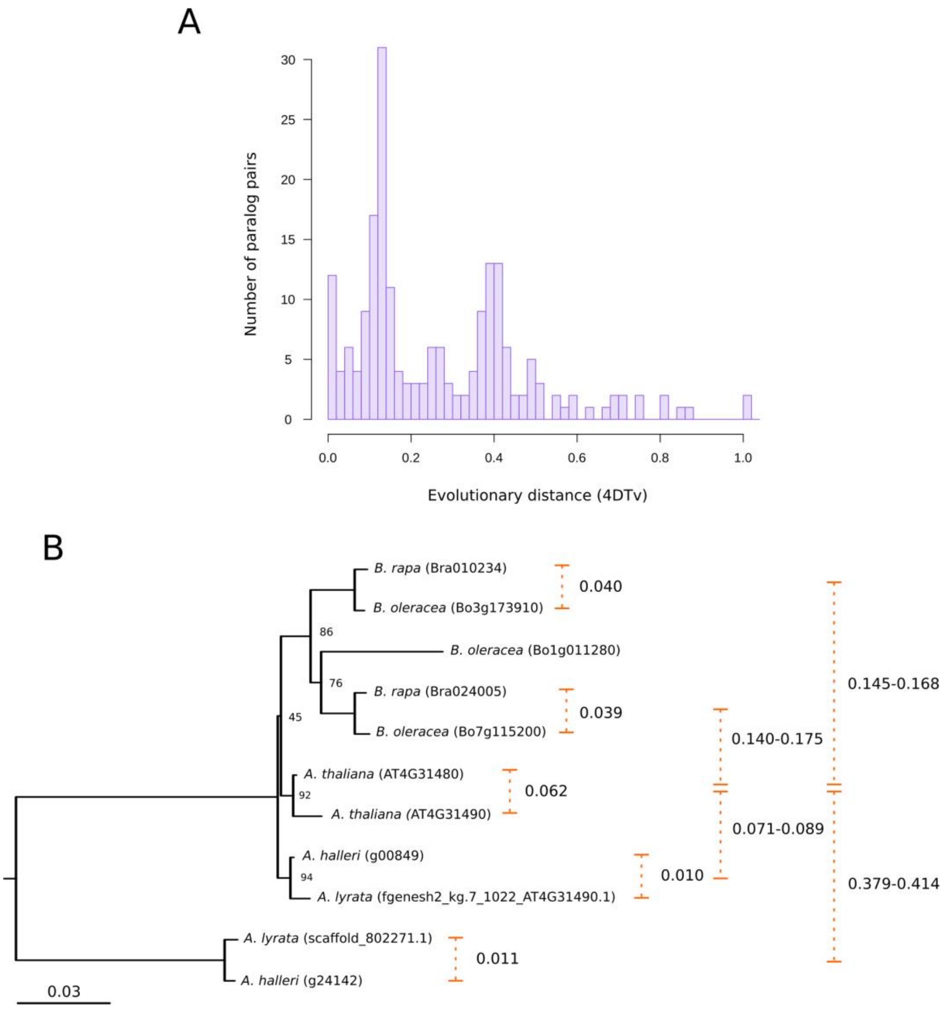

Figure S1. *β-COP* genes in plants. A. Distribution of evolutionary distances (4DTv) among paralogs within embryophytes. 4DTv pairwise distances among paralogs within species was calculated for 72 different species. **B.** Rooted maximum likelihood tree of protein sequences of several species of *Brassicaceae* family. Bootstrap value for nodes with less than 100 is shown. 4DTv distances among different homologs is shown. In the *Brassicaceae* family one of the β-COP paralogs of *A. halleri* and *A. lyrata* diverged first that the rest of the homologs in *Brassica genus* and *A. thaliana*. The 4DTv distance between the *A. thaliana* and *A. halleri* and *A. lyrata* range about 0.379 to 0.414, which coincides with a wide genome duplication (*Arabidopsis* α-WGD) shared by most *Brassicaceae* (Tiley et al., 2016). Thus, after this duplication, one of the copies was retained in both *A. halleri* and *A. lyrata* but was lost in *B. rapa*, *B. oleracea* and *A. thaliana*. The other copy of the gene suffered posterior duplications, one shared by *B. rapa*, B. oleracea at a 4DTv distance of about 0.04, posterior to the separation with *A. thaliana* (4DTv ~ 0.16), and another in *A. thaliana* (4DTv = 0.062) shortly after the separation of *A. halleri* and *A. lyrata* (4DTv = 0.071-0.089), which took place 13 Ma ago.

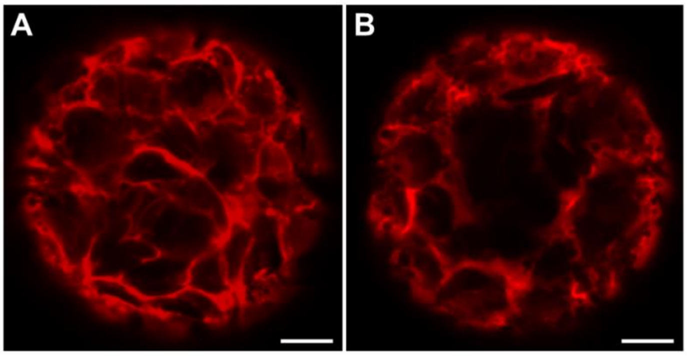

Figure S2. Localization of an ER marker protein in the *amiR-β1/β2-cop* mutant. Transient gene expression of Calnexin-RFP in *Arabidopsis* protoplasts obtained from wild type (Col-0) (A) or *amiR-β1/β2-cop* (B) plants grown 4 weeks in soil. Scale bars: 5 µm.

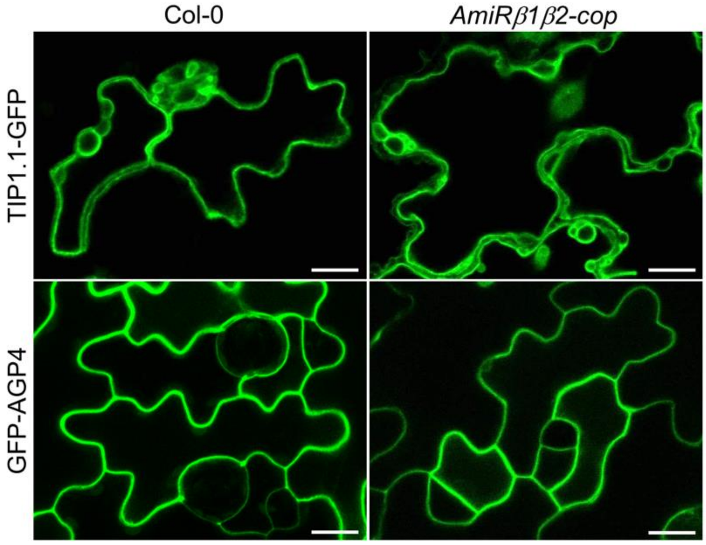

Figure S3. Localization of organelle marker proteins in the *amiR-β1/β2-cop* mutant. Transient gene expression of TIP1.1-GFP and GFP-AGP4 in *Arabidopsis* seedlings of wild type (Col-0) or *amiR-β1/β2-cop*. Scale bars: 10 µm.
